# Assessing the stability of Cognitive Stimulation Therapy’s effectiveness on dementia across US and Hong Kong

**DOI:** 10.1093/geroni/igaf122.3541

**Published:** 2025-12-31

**Authors:** Ebow Nketsiah, Marla Berg-Weger, Max Zubatsky, Allison Gibson, Jinmyoung Cho, Cara Wallace

**Affiliations:** Florida Atlantic University, Boca Raton, Florida, United States; Saint Louis University - St Louis, MO, St. Louis, Missouri, United States; Saint Lewis University, Saint Louis, Missouri, United States; Saint Louis University, Saint Louis University, Missouri, United States; Saint Louis University, Saint Louis University, Missouri, United States; Saint Louis University, Saint Louis, Missouri, United States

## Abstract

Cognitive Stimulation Therapy (CST) has demonstrated effectiveness in improving cognition and quality of life for people with mild to moderate dementia and is recognized as a cost-effective psychosocial intervention worldwide. Since its development in the United Kingdom over twenty years ago, CST has been implemented in more than thirty countries across North America, Africa, Asia, Australia, and Europe. Despite this wide adoption, few studies have examined CST’s cross-cultural effectiveness. This study explores the stability of CST outcomes across the United States (US) and Hong Kong (HK). Paired sample t-tests and effect size calculations (Cohen’s d) were used to assess CST’s impact on cognition, quality of life, depression, and physical function among persons with dementia. Findings revealed significant improvements in cognition among CST-US (t(261) = 7.41, d = .458) and CST-HK participants (t(204) = -3.13, d = -.218). Quality of life improved significantly in the US group (t(199) = 8.71, d = .616), while depression significantly decreased in both the US (t(254) = -6.35, d = -.397) and HK (t(236) = -3.08, d = -.200) groups. Physical function improved significantly in the US (t(33) = -2.06, d = -.344) but not in HK (t(84) = -.89, p = .188). These results suggest that CST is effective across cultural contexts, though the magnitude and significance of outcomes vary. The findings highlight the importance of considering cultural and contextual factors when implementing CST globally and support further investigation into adapting interventions to optimize their effectiveness across diverse settings.

